# First line antiretroviral treatment failure and its association with drug substitution and sex among children in Ethiopia: systematic review and meta-analysis

**DOI:** 10.1038/s41598-022-22237-6

**Published:** 2022-10-31

**Authors:** Seteamlak Adane Masresha, Gedefaw Diress Alen, Atitegeb Abera Kidie, Amanuel Addisu Dessie, Tadesse Mamo Dejene

**Affiliations:** 1 MPH in Reproductive Health, Woldia University, 400, Weldiya, Ethiopia; 2 MPH in Epidemiology, Debre Markos University, Debre Markos, Ethiopia; 3 MPH in Biostatistics, Woldia University, Weldiya, Ethiopia; 4 Master of Public Health, Woldia University, Weldiya, Ethiopia; 5 MPH in Reproductive Health, Debre Berhan University, Debre Markos, Ethiopia

**Keywords:** Immunology, Diseases, Health care

## Abstract

Antiretroviral Treatment (ART) has significantly decreased HIV-related morbidity and mortality among children despite the issue of drug resistance and subsequent treatment failure appearing as a challenge. Different studies have been conducted in Ethiopia regarding the prevalence of first-line ART failure among children but the magnitudes of these studies were inconsistent and had great variability. This review aimed to estimate the pooled prevalence of first line ART failure among children and its association with drug substitution and sex of children among first-line ART users in Ethiopia. The review was conducted using both published and unpublished studies until September 2020 in Ethiopia. MEDLINE, PubMed, Hinari, Web of Science, Google Scholar, Africa journal online (AJOL), Open gray literature, and online repository articles were searched. The quality of individual studies was assessed by Joanna Briggs Institute's (JBI) critical appraisal checklist. The statistical analysis was done by STATA-14 software and a random effect model was used. Heterogeneity was assessed using forest plot Cochrane Q–test and I-squared statistic. Publication bias was checked by using a funnel plot and Egger’s and Begg’s statistical tests. The interpretation was made by an odds ratio and with their respective 95% confidence intervals. The heterogeneity rate was 90% and Begg’s and Egger’s for publication bias were insignificant with p-values of 0.89 and 0.11 respectively. The pooled prevalence of pediatric first line ART failure in Ethiopia was 14.98% (95% CI 11.74, 18.21). Subgroup analysis showed that the highest failure rate was virological (9.13%). Female children had 1.4 times more risk of first-line ART failure (OR = 1.42; 95% CI 1.08, 1.85). First-line ART failure among children in Ethiopia is considerably high. Being female increases the likelihood of facing first line ART failure. More attention should be given to female children.

## Introduction

Acquired Immunodeficiency Syndrome (AIDS), a disease of no cure, can take many years to be developed following Human Immunodeficiency Virus (HIV) infection. Globally 38 million people are affected by HIV. In 2019 nearly 53% of children infected with HIV globally were receiving antiretroviral treatment (ART)^1^. More than 2 million children worldwide are infected with HIV and 90% of them live in sub-Saharan Africa^2^. In Ethiopia, there is a significant pediatric HIV-1 burden with approximately 65,100 infected children, with an estimated 3200 AIDS-related child deaths occurring annually^3^ and nearly 60% of children under the age of 15 years living with HIV were on treatment in 2018^4^.

Highly Active Antiretroviral Treatment (HAART) significantly decreased HIV-related morbidity and mortality^3^ intending to reduce the viral load to an undetectable level for further reduction of the risks of HIV transmission in addition to its role to live longer with healthier lives^5^. Many studies have reported the success of highly active antiretroviral therapy (HAART) in improving clinical and immunologic outcomes of children. However, the issue of drug resistance and subsequent treatment failure appears as a challenge^6,7^.

According to the World Health Organization (WHO) definition treatment failure (TF) could be clinical, immunological, and virological failure (VF)^8^. Virological failure is widely considered the criterion standard to detect treatment failure. Treatment failure rates of 10–34% were observed among children after 2–3 years of ART^7^. Viral load testing is a more sensitive and early indicator of TF^9^.

In Ethiopian clinical failure may be diagnosed if there is a new or recurrent clinical event indicating WHO clinical stage IV condition OR WHO clinical stage III conditions with pulmonary Tuberculosis (TB) and severe bacterial infections whereas Immunological failure is recorded if CD4 count at or below 250 cells/mm^3^ following clinical failure Or Persistent CD4 levels below 100 cells/mm^3^ and VF is defined as having Viral load above 1000 copies/mL based on two consecutive viral load measurements after 6 months of treatment start^9^.

Different factors contributed to the existence of TF like patients who didn’t change ART regimens, poor medication adherence, not taking Isoniazid (INH) prophylactic therapy, being on Zidovudine (AZT) based regimen, having lower baseline CD4 count, being bedridden during ART initiation, older age, Presence of WHO disease stage III/IV, history of injection drug use, previous protease inhibitor use, being on a second-line ART regimen, TB co-infection^10–15^. The main reasons for treatment modification in Ethiopia were toxicity, comorbidity, pregnancy, and treatment failure^16^.

Different studies have been conducted in Ethiopia to determine the prevalence of first-line ART failure among children but the magnitudes of these studies were inconsistent and characterized by great variability^3,6,17–20^. This review is conducted to fill the gaps regarding the problem of pediatric treatment failure and reduces the variability of results which was reported by individual studies. This study aimed to provide better estimates and greater power by assessing relationships that exist between first-line anti-retroviral treatment failure and its association with drug substitution and the sex of children.

## Methods

### Study setting and period

This review evaluated the relevant studies conducted in Ethiopia by using articles published until September 2020. Ethiopia is located in the Horn of Africa and has ten regional states and two administrative cities.

### Searching strategies

Both published and unpublished research articles conducted in different parts of Ethiopia focusing on HIV/AIDS treatment failure among children below the age of 18 years were searched using different searching techniques. PubMed/MEDLINE, Scopus, HINARI, Google scholar, AJOL, and Google were used as the main database for published articles. Institution repositories/libraries and research gate were used for searching unpublished studies. The advanced search for PMC was (First Line) OR Anti-Retroviral [MeSH Terms]) OR ART) OR Highly Active Antiretroviral) OR HAART) AND Treatment [MeSH Terms]) AND Failure) AND Children [MeSH Terms]) OR Pediatrics population) AND Ethiopia. Additionally, articles were searched by using keywords and phrases like “HIV/AIDS treatment Failure among Children in Ethiopia”, “Clinical Failure in Ethiopia”, “Immunological failure among Children in Ethiopia”, “Virological failure in Ethiopia”, Highly Active Antiretroviral treatment failure in Ethiopia” by using “AND, OR” bulletin. Searching for additional sources by using the reference lists of accessed articles was performed.

### Inclusion and exclusion criteria

All observational studies (Cross-sectional, case–control, and Cohort) measuring ART failure among children in Ethiopia by using clinical, immunological, or virological criteria were included. All searches were limited to the English language and studies published from 2003 when Ethiopia started ART to September 2020. Studies determining ART failure by involving both children and adults at a time were excluded.

### Measurement of the outcome variables

first line ART failure (outcome of interest) was estimated by using the national clinical, immunological, and virological criteria. Clinical failure is defined as a new or recurrent clinical event indicating WHO clinical stage IV condition OR WHO clinical stage III conditions with pulmonary Tuberculosis (TB) and severe bacterial infections whereas Immunological failure is recorded if CD4 count at or below 250 cells/mm^3^ following clinical failure Or Persistent CD4 levels below 100 cells/mm^3^ and VF is defined as having Viral load above 1000 copies/mL based on two consecutive viral load measurements after 6 months of treatment start^9^.

### Study selection and data collection

All studies identified through searching different databases were managed by using Endnote version X8 software. Duplicated studies were removed and the full text of the articles was searched by Endnote software and manually. Authors evaluated the articles by using their titles and abstracts in first phase and full text in the final phase for inclusion, and data were extracted by two independent authors.

### Quality assessment of individual studies

Two reviewers (SA & AA) independently assessed the quality of individual studies by using the JBI critical appraisal checklist for cross-sectional, case–control, and cohort studies. The tool is freely available at https://jbi.global/critical-appraisal-tools. The authors used different quality appraisal checklists for each study. On the critical appraisal process, 5 or more scores in the JBI criteria were considered to have good quality. Discrepancies in the quality assessment were resolved through a third author (GD).

### Data extraction and management

Two authors extracted the data by using the First author's name, year of publication, study design; sample size, studied population, outcomes of interest, study area, and response rate.

### Statistical analysis

The extracted data were exported to STATA/SE version 14.0 software for analysis. A descriptive summary of the included studies was presented. The pooled level of ART failure among children and its association with child sex and drug substitution was determined by using the random-effects model^21^. Since the studies retrieved were heterogeneous by study area, sample size, design, population, and study period, we declared to use a random effect model. The statistical heterogeneity was checked subjectively by using forest plot, and objectively by Cochrane Q-test and _I_^2^ statistics^22^ Subgroup analysis was carried out by region of studies. The presence of publication bias was checked by using a funnel plot and Egger’s and Begg’s statistical tests^23^. An odds ratio with a 95% confidence interval was used.

## Results

### Description of the studies

**Figure 1:**
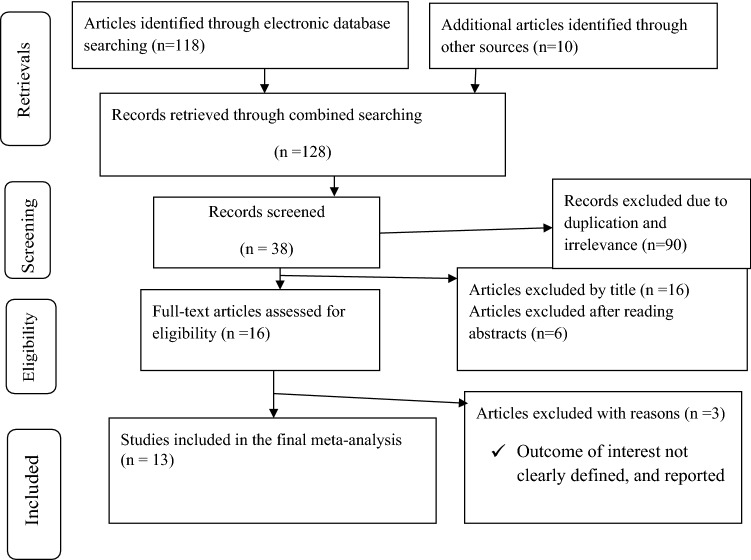
PRISMA flow diagram showing search results for the inclusion of studies focusing on first line ART failure among Children in Ethiopia, 2020.

The authors retrieved a total of 128 retrievals by using different search engines. From a total of 16 full-text articles assessed, we rejected 3 of them because the outcome of interest was not clearly defined and reported; and because the estimates were different from the outcome of interest. Finally, 13 studies were supposed to be eligible for this review (Fig. 1).

### Characteristics of original studies

A total of 13 studies having a quality scale of 5 or more by the JBI critical appraisal criteria were included for this study. Eleven of the included studies were cohort and retrospective in nature, while the rest were cross-sectional. The included studies had sample sizes that ranged from 98 to 1186. The studies were conducted from 2009 to 2020. Individual studies showed that first line ART failure among children in Ethiopia Ranges from 5.45 to 23.76%. In this meta-analysis, a total of 4931 children under the age of 18 years who were on first-line ART were included. However, there were no included studies from Somali, Harari, Afar, Gambella, and Dire Dawa regions which fulfill the inclusion criteria (Table 1).

**Table 1:** Characteristics of original studies on first line ART failure among children in Ethiopia, 2020.

ID	First author name/publication year	Region	Study design	Studied population	TF prevalence	JBI score
1	Bacha et al./2012^17^	Addis Ababa	Retrospective follow-up	1186	14.08	8
2	Brhane et al./2020^24^	Amhara	Cross sectional	238	–	6
3	Sisay et al./2018^19^	Amhara	Retrospective follow-up	824	7.65	10
4	Getaneh Y et al./2019^25^	Multi Center	Prospective and Retrospective follow-up	536	17.35	9
5	Haile et al./2019^26^	Addis Ababa	Retrospective follow-up	318	22.64	7
6	Yassin et al./2017^27^	Oromia	Retrospective follow-up	269	18.96	8
7	Sibhat et al./2020^18^	Tigray	Retrospective follow-up	404	23.76	8
8	Sorsa et al./2018^20^	Oromia	Retrospective follow-up	183	17.49	6
9	Tadesse et al./2019^28^	Multi Center	Prospective follow up	110	5.45	10
10	Osman et al./2020^29^	Oromia	Cross sectional	140	11.43	6
11	Workneh et al./2009^30^	Oromia	Retrospective follow-up	96	11.46	7
12	Yihun et al./2019^2^	Amhara	Retrospective follow-up	402	12.19	9
13	Zeleke/2016^6^	Amhara	Retrospective follow-up	225	18.22	9

*TF-* Treatment Failure.

**–** denotes no estimation due to lack of information from the original studies.

### Prevalence of first line ART failure among children

**Figure 2:**
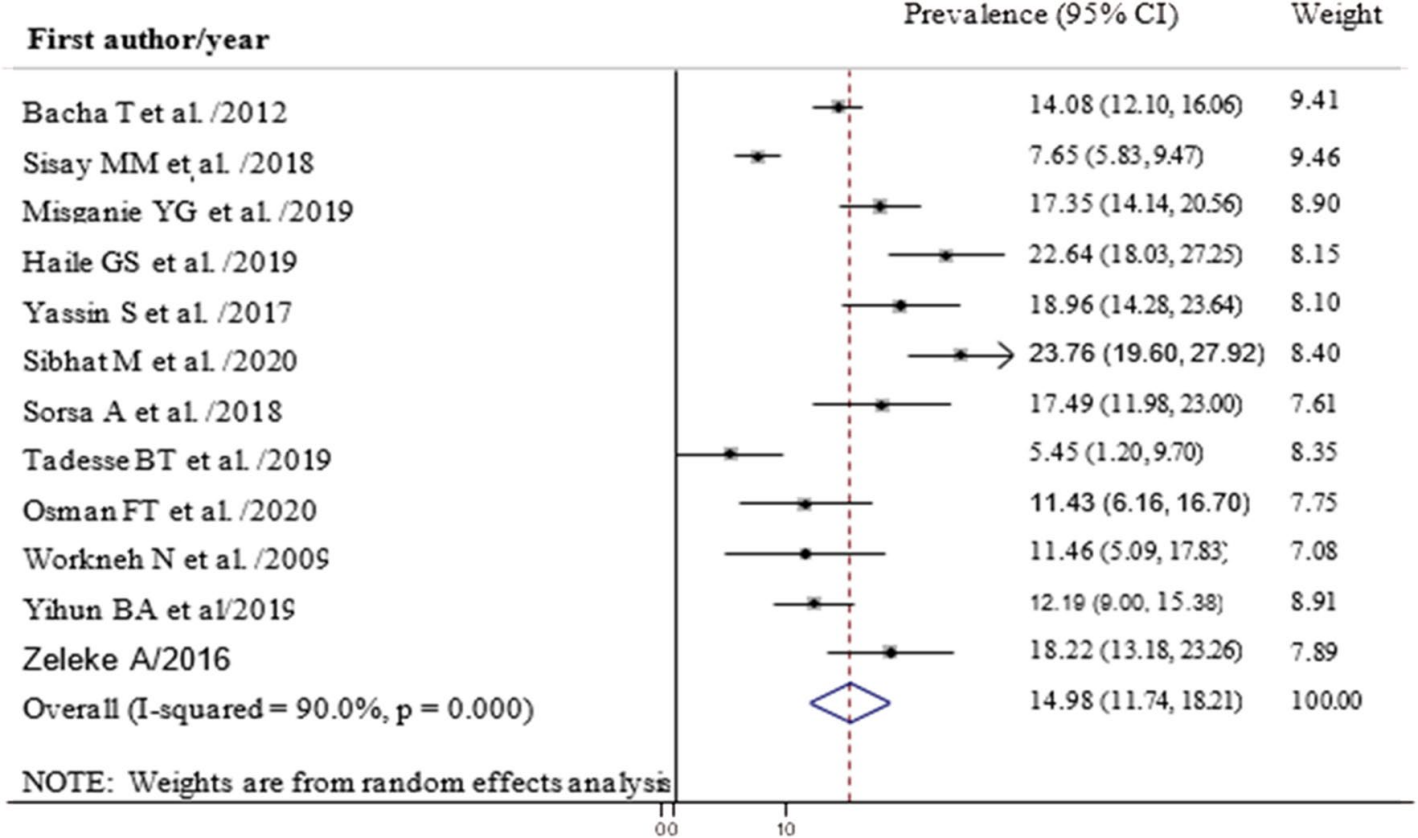
Forest plot showing the prevalence of anti-retroviral treatment failure among children in Ethiopia, 2020.

**Figure 3:**
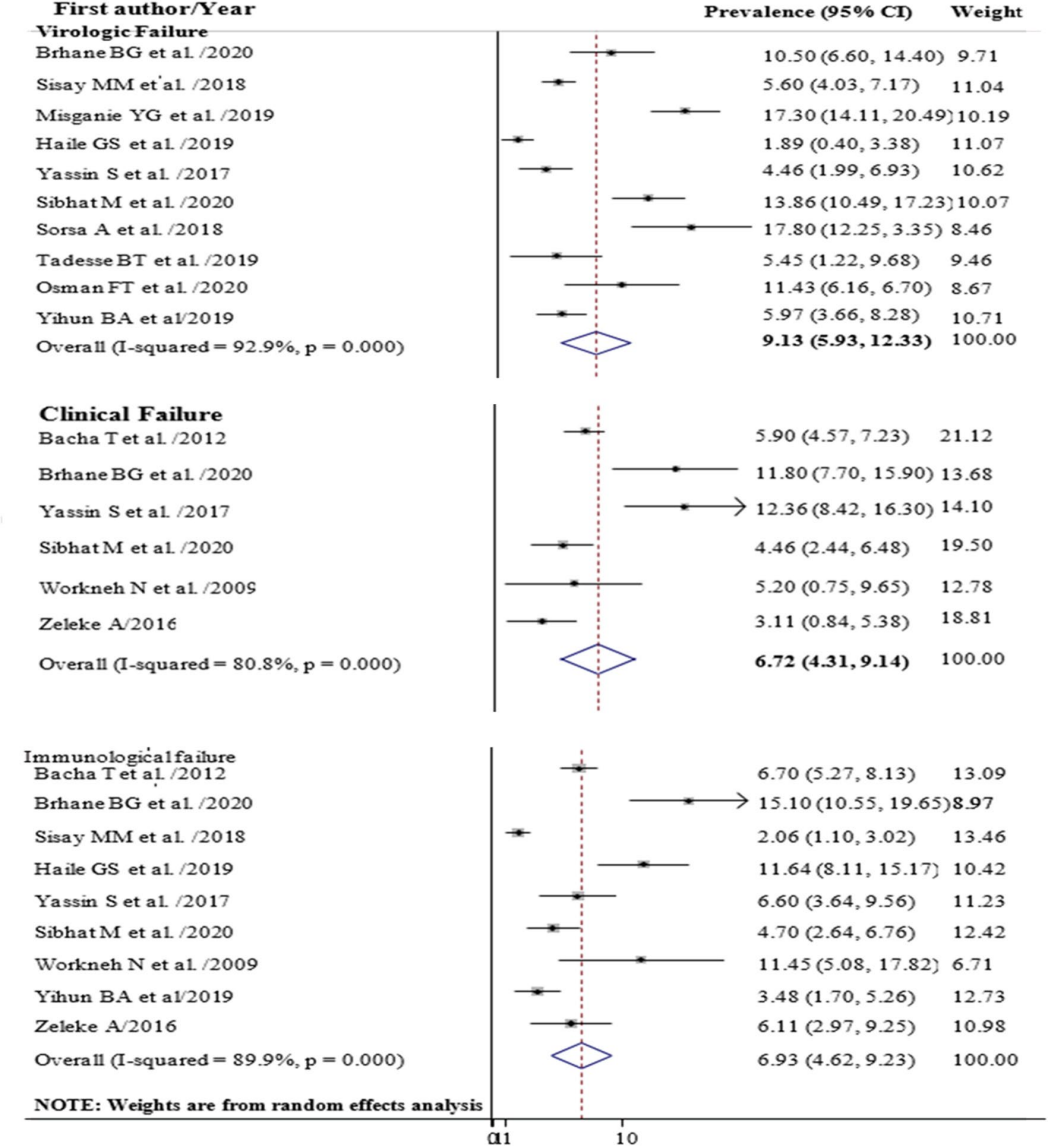
Forest plot showing the prevalence of virologic, clinical and immunological treatment failure among children in Ethiopia, 2020.

The pooled prevalence of first line ART failure among children in Ethiopia was 14.98% (95% CI 11.74, 18.21) with high heterogeneity between studies (I-squared = 90.0%) (Fig. 2). Subgroup analysis showed that the highest failure was Virological (9.13%) followed by Immunological (6.93%) and clinical failure (6.72%) (Fig. 3). Analysis by region indicated that the highest virological treatment failure rate was recorded in Tigray (13.86%) and Oromia (10.95%) (Table 2).

**Table 2:** Subgroup analysis by region showing the prevalence of clinical, immunological and VF among children in Ethiopia, 2020.

Subgroup analysis (by region)	Overall TF prevalence (95%CI)	CF prevalence (95%CI)	IF prevalence (95%CI)	VF prevalence (95%CI)
Addis Ababa	18.10 (9.72, 26.47)	5.90 (4.57, 7.23)	8.90 (4.09, 13.71)	1.89 (0.40, 3.38)
Amhara	12.27 (6.78, 17.76)	11.80 (7.70, 15.90)	6.03 (2.42, 9.64)	6.76 (4.49, 9.04)
Oromia	15.07 (11.11, 19.03)	8.86 (1.84, 15.87)	8.17 (3.72, 12.62)	10.95 (2.76, 19.13)
Tigray	23.76 (19.61, 27.92)	4.46 (2.44, 6.48)	4.70 (2.64, 6.76)	13.86 (10.49, 17.231

CF clinical failure, *IF* immunological failure, *VF* virologic failure, *TF* treatment failure.

### Factors associated with first line ART failure

**Figure 4:**
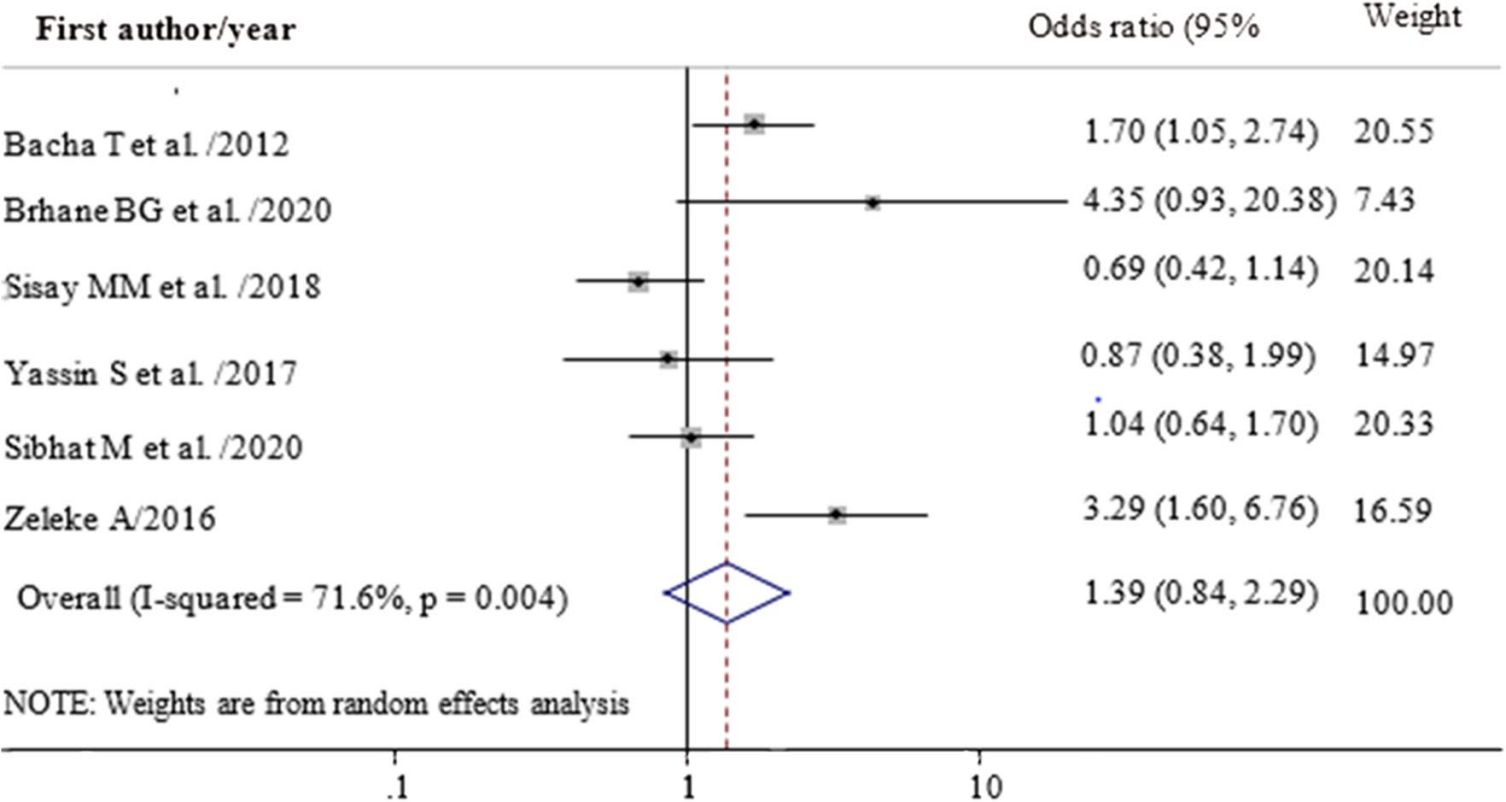
Forest plot of adjusted odds ratio showing the association between having drug substitution and treatment failure among children in Ethiopia, 2020.

**Figure 5:**
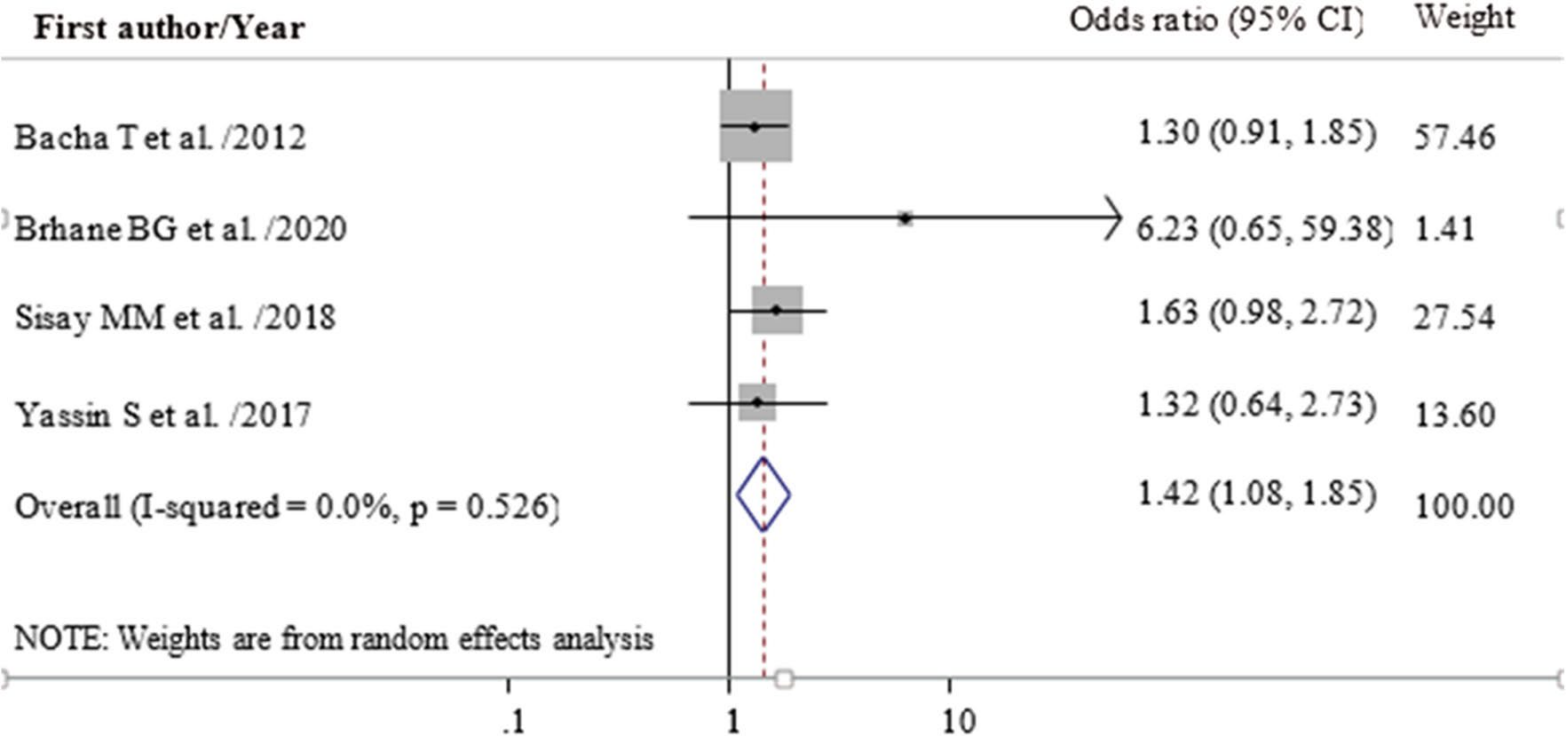
Forest plot of adjusted odds ratio showing the association between being female children and ART failure among children in Ethiopia, 2020.

In this meta-analysis, we examined the association between drug substitution and the sex of children with first line ART failure. Accordingly, the odds of first line ART failure were 1.4 times higher among children who had a history of drug substitution (OR = 1.39; 95% CI 0.84, 2.29) (Fig. 4). Female children had an increased risk of first line ART failure (OR = 1.42; 95% CI 1.08, 1.85) as compared to their counterparts (Fig. 5).

### Publication bias

**Figure 6:**
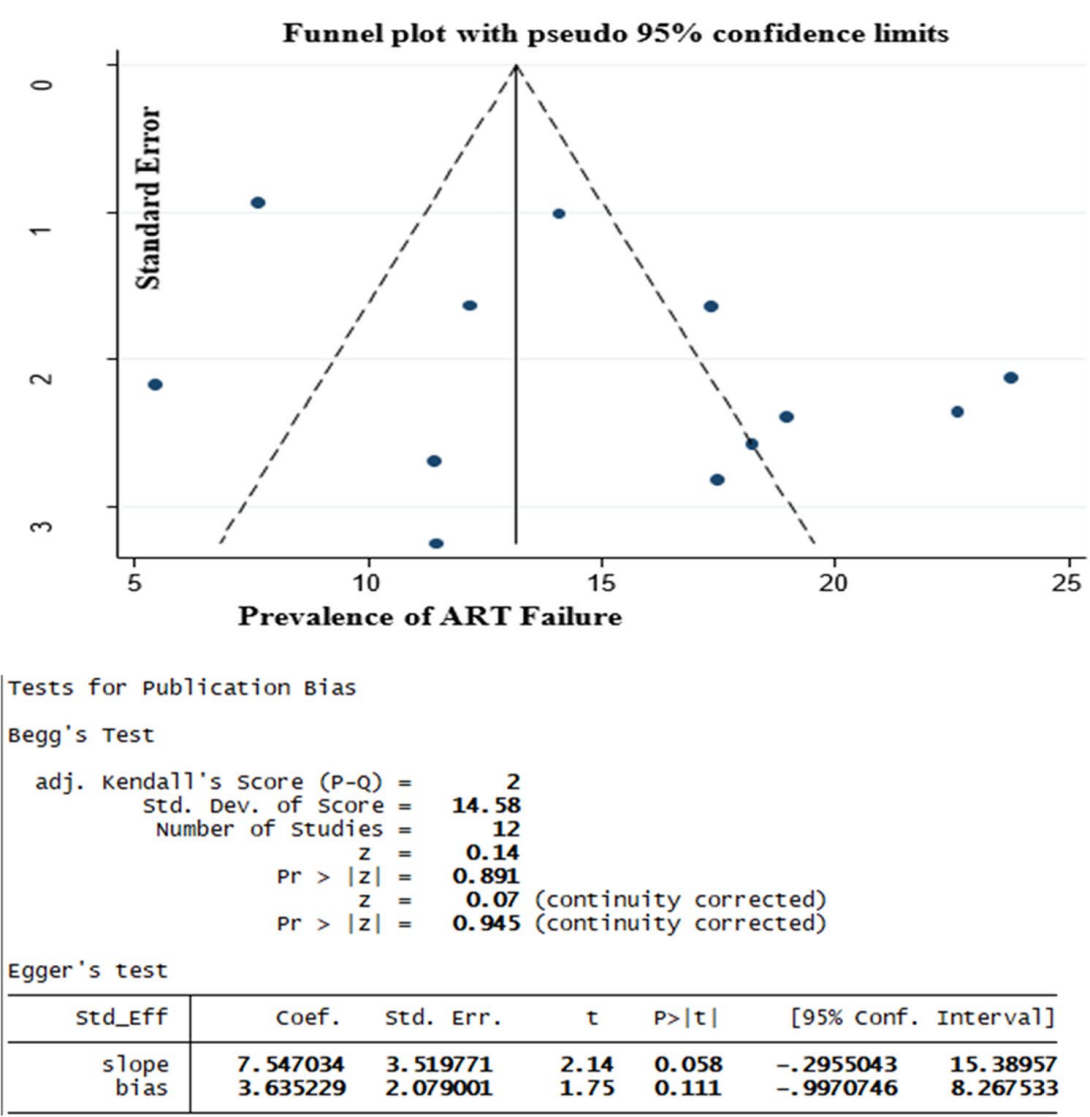
Test of publication bias for prevalence studies by using Begg’s and Egger’s test, 2020.

Begg’s and Egger’s test showed that there was no publication bias (Fig. 6).

## Discussion

Based on the results of this review the pooled prevalence of first-line ART failure among children in Ethiopia was 14.98%. The result was closer to previous studies conducted in Ethiopia at the general population level which were 15.9%^31^ and 15.3%^32^. This review showed that the magnitude of VF among children was 9.13% which was lower than studies conducted in the USA (40%)^33^, India (29%)^34^, Iran (29.2%)^35^ South Africa (16.9%)^36^. Tanzania (34%)^37^, Zambia (40%), and Senegal (64%)^38^. The possible reasons for the lower magnitude in this review may be associated with the difference in defining VF which was more than 1000 copies/ml in the current review, while more than 400 copies/ml were considered as VF in the above studies^36^, the differences in the populations to be studied whereby adults and children were included and the use of virologically failed children to estimate VF after some point in time^33^; and the difference in the types of HIV infections whereby dual HIV infections (Infection by both HIV-1 and 2) become a problem in west African countries^38^. The presence of a high level of ART adherence in Ethiopia which was more than 93%^32–41^ than studies conducted in Tanzania (65.3–84%)^42,43^, Uganda (79%)^44^ and Nigeria (65.6–91%)^45,46^ may contribute to the low level of TF in this study.

According to the results of this review being female was 1.4 times more likely to have first line ART failure even though a study conducted in Gondar showed that being male had higher odds of ART failure (AOR = 3.15)^47^. The result was consistent with the findings of previous studies in the country^11^ though, it was slightly higher than a study conducted in Addis Ababa^17^. The possible reason for this variation could be attributed to our review result was the pooled estimate from many studies. The highest risk of delaying ART initiation and experiencing more side effects among females, and the biological differences existed in metabolizing ART drugs between females and males^48,49^ might contribute to the high rate of first line ART failure among female children. Studies also indicated that female children had a higher risk of severe anemia^50^ and being low weight^51^ which may further affect their level of immunity and could contribute to the rapid progression of disease stages.

Regarding drug substitution, even though it was not statistically significant, first-line ART drug substitution increased the risk of ART failure (OR = 1.39; 95% CI 0.84, 2.29). This finding was supported by studies conducted in Ethiopia and Myanmar^6,32,47,52^. The possible reason may be due to the availability of limited facilities for viral load testing in resource-limited countries like Ethiopia, forced to use clinical and immunologic criteria, which have low specificity and positive predictive values, to diagnose TF and then to substitute or change drugs may result in unnecessary switches/substitutions of ART drugs^53^. Drug substitution/change in Ethiopia may be a result of problems in the supply system or client-related factors without consideration of risks of drug resistance before changing a particular regimen^54^ which may fail. This highlights the need for a drug resistance test before switching/substituting ART drugs.

## Strength and limitations

The use of internationally accepted critical appraisal tools and searching for the inclusion of unpublished data was the strength. The retrieved studies represent only four of the twelve regions within the country so the finding may not represent the nation. High heterogeneity within the included studies was detected in this analysis. The criteria’s used to determine first-line ART failure across the included studies were different, which may under or overestimate the prevalence.

## Conclusion

First-line ART failure among children in Ethiopia is considerably high. Being female increases the likelihood of facing ART failure. More attention should be given to female children.

## Data Availability

The data set used and analyzed for the review is available from the corresponding author upon reasonable request.

## Abbreviations

AIDS: Acquired immunodeficiency syndrome
ART: Antiretroviral treatment
HAART: Highly active anti-retroviral treatment
HIV: Human immunodeficiency virus
TB: Tuberculosis
TF: Treatment failure
VF: Virological failure
WHO: World Health Organization
